# Applying the Dual Filial Piety Model in the United States: A Comparison of Filial Piety Between Asian Americans and Caucasian Americans

**DOI:** 10.3389/fpsyg.2021.786609

**Published:** 2022-02-03

**Authors:** Amy J. Lim, Clement Yong Hao Lau, Chi-Ying Cheng

**Affiliations:** ^1^Discipline of Psychology, College of Science, Health, Engineering and Education, Murdoch University, Murdoch Singapore, Singapore, Singapore; ^2^School of Social Sciences, Singapore Management University, Singapore, Singapore

**Keywords:** filial piety, Dual Filial Piety Model (DFPM), Asian American, Caucasian American, collectivism

## Abstract

The definition and measurement of filial piety in existing research primarily focuses on the narrow conceptualizations of Asian filial piety, which would inflate cultural differences and undermine cultural universals in how people approach caring for their elderly parents. Employing the Dual Filial Piety Model (DFPM), this study aimed to examine the relationship between filial piety and attitude toward caring for elderly parents beyond the Asian context. In our study (*N* = 276), we found that reciprocal filial piety (RFP) does not differ across cultures while authoritarian filial piety (AFP) does. We also found that collectivism, rather than ethnicity, predicted RFP and AFP, which in turn predicted positive attitude toward caring for elderly parents. Our work demonstrates the cross-cultural applicability of the DFPM and highlights the universal and culture-specific aspects of filial piety.

## Introduction

Increased life expectancies and declined fertility rates have led to global population aging, and they are expected to continue at an accelerated rate (United Nations Department of Economic and Social Affairs Population Division, 2019). The number of persons aged 65 and above has outnumbered the population of children aged five and under since 2018 (United Nations Department of Economic and Social Affairs Population Division, 2019). By 2050, one in six people will be aged 65 and above, up from 1 in 11 people in 2019 (United Nations Department of Economic and Social Affairs Population Division, 2019). As this demographic trend holds significant implications for labor force participation, economic growth, and consumption patterns (Bloom and Luca, 2016), managing the needs for an aging population is a pertinent social concern in the twenty-first century. Amongst the strategies and action plans to manage population aging, the provision of elder care constitutes a major portion of the list (World Health Organization, 2019). More critically, provision of care from family systems is increasingly important to aid the financial sustainability of public elder care services in coping with the soaring demands and enhancing quality of care.

With the moral underpinnings filial piety provides for parent-child relations, extant research has examined the impact filial norms have on the support and care for elderly parents. However, despite evidence suggesting that filial responsibilities are also observed in Western cultures, most of the existing research on filial piety has primarily focused on Asian cultures. The indigenous conceptualizations of filial piety are likely to inflate cultural differences and undermine cultural universals in how people approach caring for their elderly parents. In this paper, we contend that filial piety is more universal than culturally exclusive—filial piety behavior is also manifested in non-Asian societies. Using the DFPM framework, we intend to demonstrate that filial responsibilities can be observed across cultures, and that it influences filial attitudes and behaviors.

### Filial Piety and Care for Elderly Parents

Traditionally, elders in Asian societies are revered. This stems from the emphasis on Confucian values in Asian societies, particularly that of its central tenet—filial piety. Filial piety is typically regarded as the duty of adult children to care for their elderly parents (Cicirelli, 1993; Sung, 1995; Leichtentritt et al., 2004); beyond the act of providing care, it constitutes respecting, honoring, and obeying one's parents (Lee and Mjelde-Mossey, 2004). The fulfillment of filial duties includes preserving family harmony, being affectionate toward parents, having a sense of responsibility toward parents, minimizing the worries of parents, repaying the physical and financial sacrifice parents has made, and even staying close to parents or living together with them (Sung, 1995; Ho, 1996; Kao and Travis, 2005). It is a high virtue and dominant social norms in the majority of Asian societies including China, Taiwan, Japan, Korea, Thailand, Vietnam, Singapore, Malaysia, Indonesia, India and Bangladesh (e.g., Wangmo, 2010; Nichols, 2013). As a way of demonstrating filial piety, empirical evidence shows that the percentage of intergenerational co-residence in East Asian societies (i.e., China, Taiwan, Japan, and Korea) was well within 23 and 47% (Lin and Yi, 2013). Higher rates of adult children living together with their older parents was also found in Japan, compared to the U.S. (Levy et al., 2009; Nauck and Ren, 2021).

The virtue of filial piety facilitates intergenerational relationships (Yeh, 2003), and encourages care and support of elderly parents (Lai, 2007; Khalaila and Litwin, 2011). Support toward elderly parents involves the dependency of elderly parents on adult children (Chen, 2006). These intergenerational support behaviors include co-residence, as well as support provision from adult children (Yi and Lin, 2009). Multigenerational co-residence is characterized by the three-generational family structure, where adult children, with their (young) children, and the aging parents live together. It helps adult children to fulfill the expected filial duties to their elderly parents because it provides the opportunity for adult children to be the caregivers (Chen, 2006); this allows them to demonstrate their affection, obedience, and commitment toward their elderly parents (Lin and Yi, 2013). Indeed, endorsement of filial norms is positively associated with rate of co-residence—adult children who are in favor of filial responsibilities were more likely to live with their parents and have more face-to-face contact with them (Lin and Yi, 2013)—in East Asian societies including Japan and Korea. Adult children's filial attitudes were also found to have a positive impact on patrilocal co-residence. Particularly in Taiwan, couples with stronger filial attitudes were found to either live with their parents, or stay close to them (Chu et al., 2011). Similarly in China, filial piety is also significantly associated with the rates of adult children co-residing with their aging parents (Zhang et al., 2014). Additionally, adult children with higher filial responsibilities provide greater financial support to their aging parents (Lin and Yi, 2013). In short, greater endorsement of filial piety is associated with higher intergenerational support in East Asian context.

Additionally, studies that have examined the role of filial piety in caregiving show that filial piety is negatively associated with caregiving burden (e.g., Lai, 2007); filial piety served as a buffer against negative effects of stressors of caregiving burden (Kim and Kang, 2015). Moreover, higher levels of filial piety from caregivers also predicted more positive appraisals of caregiving to their elderly parents (Lai, 2010). Endorsements of filial piety were also associated with lower levels of distress, greater self-efficacy, as well as more positive caregiving experiences (Holland et al., 2010). These studies collectively suggest that filial piety is not only associated with actual support toward one's elderly parents, but also the positive attitudes toward caring for their elderly parents.

Notwithstanding the empirical evidence that the endorsement of filial piety and care of elderly parents are more pronounced in Asian cultures (Löckenhoff et al., 2015), intergenerational care is also observed in Western societies. For instance, 80–90% of all care to elders in the United States is provided by family members (Westbrook, 1989; Moon et al., 2017; Varadaraj et al., 2021), and more than 50% are provided by their adult children (Ornstein et al., 2017). In addition, ~75% of British elderly people receive some form of care (Henz, 2006)—from their adult children or spouses (Akgun-Citak et al., 2020). In fact, studies have shown that (non-Asian) individuals also possess positive attitudes toward caregiving of one's elderly parents. In a national survey in the United States, 57% of family caregivers (of elderly parents) described their experiences positively—such as it being rewarding, and enjoyable (National Alliance for Caregiving and American Association of Retired Persons, 1997). This trend still holds true in a more recent study (Conway, 2019). They also identified additional benefits of pride in making their elderly parents happy, of earning their gratitude, and of repaying parents. Further, the rewards and benefits of caring for their elderly parents continues to extend as the caregivers age. For instance, other than attaining greater maturity and preparedness for their own aging (Ziemba, 2002), caregivers even accrued benefits such as improvement in their relationships with their elderly parents and other family members, personal satisfaction in spending time caring for them, as well as pride in familial cooperation to meet their parents' needs (Strawbridge and Wallhagen, 1991; National Alliance for Caregiving and American Association of Retired Persons, 1997). These findings not only suggest that filial behaviors are exhibited by individuals from Western societies, they also demonstrate that Western adult children hold positive attitudes toward caring for their elderly parents. More critically, these findings attest to the likelihood that filial piety could be culturally universal—in that the dominant constructs and measures of filial piety based on Asians' filial norms (Bedford and Yeh, 2019) could partially capture the motive of Western adult children's care and support for elderly parents.

Current conceptualizations of filial piety may lead to equivocal conclusions regarding cross-cultural differences in the attitudes toward care and support for elderly parents. Given the strong emphasis of filial piety in Asian societies, it is intuitive for Asian adult children to hold more positive attitudes in caring for their elderly parents than those brought up in Western societies, where there are fewer cultural ideologies that encourage the sacrifice and care for elderly parents (Laidlaw et al., 2010). However, some studies offer contradictory evidence showing that Westerners support care of their own elderly parents more than Asians. For instance, as compared to a Japanese sample, U.S. respondents have more positive attitudes toward family obligations and were more likely to agree that children should make sacrifices to support their parents (Elmelech, 2005). Similarly, Anngela-Cole and Hilton (2009) found that Caucasian Americans felt greater obligation toward their parents and had more positive attitudes toward caregiving than Japanese Americans. Further, in a sample of family caregivers, non-Asian Americans were more likely to say that caregiving has positive (personal and familial) impact on them than the Asian Americans (Arnsberger et al., 2009). Furthermore, the history of Confucian principles in the East Asian cultures and conception of joint family system in South Asian cultures impel individuals to respect, obey, and care for *their elderly family members* (Sung, 1995; Koyano, 1996; Singh, 2005), North Americans who do not abide to Confucian principles may be compelled to love and care for their senior parents with different motives. Collectively, the current research seek to demonstrate that (1) filial piety is not necessarily unique to Asian cultures and can be observed in Western societies, and (2) existing indigenous Asian conceptualizations of filial piety may be applied beyond the Asian cultural context and can be used to detect some aspects of filial attitudes and behaviors across cultures. To this end, we employ the Dual Filial Piety Model (DFPM) in our investigation of filial piety across cultures in our paper (Yeh and Bedford, 2003; Bedford and Yeh, 2019).

### Dual Filial Piety Model

According to the Dual Filial Piety Model (DFPM; Yeh and Bedford, 2003), filial piety consists of two fundamental aspects: reciprocal filial piety and authoritarian filial piety. Reciprocal filial piety (RFP) encompasses the genuine gratitude one has for their parents' effort and sacrifice, and manifests as the voluntary support and care for one's parents (Bedford and Yeh, 2019)—where it develops from the accumulative positive interactions between parent and child. In contrast, authoritarian filial piety (AFP) manifests as the obedience and fulfillment of obligatory duties as a child to one's parents and develops through the fulfillment of the expectations one's parents hold of them (Bedford and Yeh, 2019).

Studies have demonstrated that care for one's elderly parents is ubiquitous across Asian and Western societies. Although the theory of filial piety is chiefly drawn upon Confucian teaching and research on filial piety is largely conducted in Asian context (e.g., China, Taiwan, Japan, Korea, Vietnam, India and Thailand; Nguyen, 2016; Serrano et al., 2017; Sringernyuang et al., 2020), care for one's elderly parents is also observed in Western cultures. Recent research suggests that there is similarity between the conceptualization of Asian filial piety and values in other cultures (see Bedford and Yeh, 2019). For instance, both Hispanic familism and filial piety stress social relationships over individual needs (Schwartz et al., 2010). Providing care for one's parents, family, and community is not only ingrained in Asian Americans, Hispanic Americans, as well as African Americans also share similar perspectives (Pharr et al., 2014). While Asian cultures subscribe to a cultural model that dictates a rigid taxonomy for caregiving based on one's gender and position within the family (e.g., caring for elderly parents is expected for them; Ngan and Wong, 1996), rejecting caregiving (of elderly parents) and to place the responsibility outside one's family can be seen as unacceptable even for African American, and Hispanic American caregivers (Pharr et al., 2014). Further, even in the Netherlands, it was found that the greater the perceived obligation, the greater the support one gives to their elderly people, the emotionally closer the familial relationships (Stuifbergen et al., 2008). These findings suggest that it would be erroneous to conclude that filial piety, and its implications on caring for elderly parents, is unique to Asian cultures.

The application of the DFPM in filial piety research has shown promise in uncovering the different aspects of filial piety, and its consequential effects on the care of one's parents—where previous research that relied on Indigenous Asian definitions of filial piety were not able to demonstrate. The majority of filial piety research draws upon Confucian teachings that are prevalent in Asian societies and conceptualizes the foundation of filial piety on role obligations driven by an authoritarian relationship between parents and children. With the DFPM, it is evident that RFP and AFP have distinct characteristics and assumptions of interpersonal relationship between parents and children. RFP is driven by a horizontal relationship, assuming equal relationship between two individuals, even when they are parents and children. In comparison, AFP is determined by a vertical relationship, assuming a hierarchical relationship between different family roles such as parents and children (Tsao and Yeh, 2019). Empirical findings demonstrate that RFP and AFP exhibit different effects on adult children's care for senior parents. An analysis of a nationally-representative sample of 1,463 adults in Taiwan (collected by the Taiwan Social Change Survey 2006) demonstrated that adult children's RFP has a significant positive effect on the frequency of financial support, household labor assistance, and emotional support for elderly parents, even after controlling for demographic and family structure variables (Yeh, 2009). In contrast, their AFP had a positive correspondence only with the frequency of providing household labor, and not with providing financial or emotional support for elderly parents. As such, this demonstrates that this distinction of filial piety can reveal a deeper understanding of filial attitudes and behaviors beyond the traditional conceptualizations of filial piety. More critically, this supports the employment of the DFPM as a framework to address filial piety in a Western cultural context (Bedford and Yeh, 2019; Tsao and Yeh, 2019).

### The Present Research

In this research, we propose that the concept of filial piety could be culturally universal and could be observed in Western societies such as the United States. Furthermore, we argue that cultural universality as well as cultural uniqueness in filial piety could be both observed between Asian and American cultural groups. We intend to apply the two factors of DFPM framework—RFP and AFP—to investigate the relationship between filial piety and adult children's care for elderly parents across cultures.

We argue that RFP, which develops from positive interactions between parent and child with an assumption of equal relationship between the two parties, is likely to be observed among both Asians and Americans, and as such, will not differ across cultures. The propensity to provide care for elderly parents is motivated by factors including love, affection, as well as a sense of obligation (Dean et al., 2020)—factors that are not unique to any one society. Similar to that of Asian adult children (Lin and Yi, 2013), individuals in Western cultures were found to care for their parents out of love and affection rather than indebtedness (Blustein, 1977; Dixon, 1995). As such, we propose that the reciprocal relationship between adult children and parents can be observed in both Asian and American societies and can be captured by the behavioral manifestation of RFP in DFPM framework.

In contrast, we proposed that AFP, which develops from the belief of an authoritarian relationship between parents and children, would differ across cultures, especially between Asians and Americans. The foundation of AFP is based on Confucian teachings that are guided by authoritarianism and familism, which contribute to family hierarchy and role obligations (Chien, 2016). Adult children are expected to prioritize elderly parents' needs and expectations. When facing conflicts between parents' and own desires, adult children are expected to downplay their own needs and expectations to in order to fulfill their parents' needs and expectations (Tsao and Yeh, 2019). These filial norms and practices are specific to Asian societies and are not congruent with cultural values and norms in Western societies. Therefore, we predict that AFP is likely to be observed more among Asians Americans than Caucasian Americans.

Most of the existing research that have examined cultural similarities and differences in the attitudes toward care for older adults classified cultural groups geographically by country (e.g., China vs. the United States). This practice has been questioned by scholars as it oversimplifies cultural nuances (North and Fiske, 2015). To address this shortcoming, we classify cultural groups by ethnicity in the same society in our study. Specifically, we examine the likely cultural similarities and differences between Caucasian Americans and Asian Americans who reside in the same geographical location (i.e., the United States).

Additionally, given that filial piety is largely associated with dominant behavioral norms of collectivism (Schwartz et al., 2010), a cultural value that significantly distinguishes Asian and Caucasian cultures (Hofstede, 1980; Bebko et al., 2019; Pereira et al., 2019), we look into the influence of collectivism on the two types of filial piety as well as its downstream effect on individuals' attitudes toward caring for elderly parents and their caregiving behaviors. Drawing upon Matsumoto's (1999) recommendation for cultural comparison research, the significance of cultural difference needs to be demonstrated by identifying the potential contextual variables such as personal values and tendencies that underlie the link between cultural groups and individual outcomes. Given that collectivism affords the maintenance of interpersonal ties and emphasis on a collective identity, we further proposed that cultural value of collectivism (Hofstede, 1980) serves as the underlying cultural dimension for filial piety. Specifically, as collectivism facilitates an interdependent self that often blurs one's self-boundary from significant others including parents (Markus and Kitayama, 2010), individuals with a high level of collectivism will exhibit more RFP. This is because the children's self is closely knitted with their parents both psychologically and socially and possibly see their parents as the extension of their self just like how their parents see them (Markus and Kitayama, 1991; Fu and Markus, 2014). As a result, individuals high on collectivism are compelled to exhibit RFP and reciprocate their parents' love and support for them. In addition, endorsement of collectivism will also facilitate adult children's AFP. Asian cultures largely informed by Confucian teachings breeds collectivism, familism, and social hierarchy (Yu and Yang, 1994). Individuals in a collectivistic culture pursue social harmony that involves sacrificing self for the group and family (Yu and Yang, 1994). Corresponding to the Confucian teachings, collectivistic parents with a predilection for hierarchy and authoritarian moralism have the legitimacy to discipline their children and these children are taught to submit to their subordinate position within the family (Bejanyan et al., 2015; Wu and Chen, 2021). Given that AFP stems from one's adherence to role obligations based on family hierarchy (Tsao and Yeh, 2019)—with children looking up to their parents as possessing absolute authority (Bedford and Yeh, 2019), children who endorse the collectivistic value may also feel duty-bound to care for their parents as a way of showing gratitude for raising them—beyond the affection for their parents. In sum, we predict that the endorsement of collectivism drives both RFP and AFP, which then predicts Asians' and American' attitudes toward caring for elderly parents and caregiving behaviors of their elderly parents.

## Methods

### Participants

A total of 276 participants were recruited through Amazon Mechanical Turk (192 females, *M*_age_ = 40.26, *SD*_age_ = 14.30)^1^. In this sample, all participants resided in the United States; 161 participants identified with White/Caucasian as their ethnicity, and 115 participants identified with Asian as their ethnicity^2^. The majority of participants (*N* = 140) reported to be working full-time as their employment status (*N*_workingpart−time_ = 44, *N*_student_ = 27, *N*_unemployed/others_ = 65).

### Procedure

Upon providing informed consent, participants completed a series of questionnaires that assessed individualistic and collectivistic values, filial piety, and their attitude toward caring for elderly parents^3^. They also responded to questions related to the behavioral interactions they have with their parents, including if they stayed with their parents, how often they spent time looking after their parents in the past year, proportion of the salary they gave to their parents, how often they visited their parents, and how often they make phone contact with them. Finally, participants provided demographic details before completing the study.

### Materials

#### Filial Piety

Filial piety was assessed with the 16-item Dual Filial Piety Scale (DFPS; Yeh and Bedford, 2003); 8-items measured Reciprocal Filial Piety (RFP) and 8-items measured Authoritarian Filial Piety (AFP). Participants indicated the extent they agreed to statements such as “Be grateful for my parents for raising me” for RFP, and “Avoid getting married to someone my parents dislike” for AFP, on a 7-point Likert scale (1 = *strongly disagree*, 7 = *strongly agree*). Items were averaged to form a single index for RFP (*M* = 5.66, *SD* = 1.21, α = 0.95) and a single index for AFP (*M* = 3.27, *SD* = 1.23, α = 0.88), where higher scores reflect higher filial piety beliefs.

#### Individualism-Collectivism

Individualistic and collectivistic values were measured with the Culture Orientation Scale (Triandis and Gelfand, 1998). Participants responded to 16 items, such as “My personal identity, independent of others, is very important to me,” and “It is important to me that I respect the decisions made by my groups” on a 9-point Likert scale (1 = *never or definitely no*, 4 = *always or definitely yes*). Items were averaged to form a single index for individualism (*M* = 5.78, *SD* = 1.11, α = 0.68) and a single index for collectivism (*M* = 6.36, *SD* = 1.34, α = 0.84), where higher scores indicated higher levels of individualistic and collectivistic values, respectively.

#### Attitude Toward Caring for Elderly Parents

To measure participants' attitude toward caring for their elderly parents, we adapted 17 items employed by Dellmann-Jenkins and Brittain (2003) in their study of filial responsibility attitudes. These items included statements such as “We should look to the children to support their elderly parents,” and “Adult children should overlook the trouble that elderly parents might cause in their home lives.” Participants responded to the items on a 4-point Likert scale (1 = *strongly agree*, 4 = *always or strongly disagree*). Items were reverse coded and averaged to form a single index for one's attitude toward caring for elderly parents (*M* = 2.74, *SD* = 0.49, α = 0.91), where higher scores indicated a more positive attitude toward caring for one's elderly parents.

#### Filial Behaviors Toward Elderly Parents

To measure participants' care behavior toward elderly parents, participants were required to respond to three items: “How often did you spend time looking after your parents in the past year” (1 = *seldom or not at all*, 6 = *from morning to night every day*), “How often do you visit your parents,” and “How often do you make phone contact with them” (1 = *daily or almost daily*, 5 = *not once over the past 12 months*). Items were standardized, and averaged to form a single index for filial behaviors (*M* = −0.16, *SD* = 0.81, α = 0.81), where higher scores indicated more frequent filial behaviors displayed toward elderly parents.

## Analytical Strategy

We first determined whether three scales used in the study (i.e., Dual Filial Piety Scale, Culture Orientation Scale, and Attitudes toward Caring for Elderly Parents) measured the same constructs in both cultures (i.e., that they demonstrated measurement invariance across the Asian American and Caucasian American samples). The measurement invariance analyses were conducted using the lavaan package (Rosseel, 2012), in the R environment (R Core Team, 2020)—using maximum likelihood estimation with robust standard errors, and a Satorra-Bentler scaled test statistic (MLM).

First, the factorial structure of each scale was assessed for the total sample. For the scales which various models had been proposed, fit indices of the models were compared. In case of model misspecifications, the cause of specific error was examined *via* modification indices. To evaluate the goodness of fit of the models, we used the following fit indices and cut-off values recommended by Hu and Bentler (1999), and Brown (2015). Root mean square of approximation (RMSEA) values smaller than 0.08 indicated a reasonable fit and values smaller than 0.05 a good fit (MacCallum et al., 1996). Comparative fit index (CFI) values >0.9 indicated a good fit (Bentler, 1990). Standardized root-mean-square residual (SRMR) values smaller than 0.08 indicated a good fit (Kline, 2016).

Second, we determined the three scales' cross-cultural equivalence through multi-group confirmatory analyses (MGCFA)—by measurement invariance (MI) testing—that includes a series of model comparisons. Three consecutive models were estimated, with each serving as a basis for comparison to the preceding model. At each comparison step, equality constraints were added in addition to the previous models (Steenkamp and Baumgartner, 1998). In cross-cultural research, three levels of measurement invariances are usually estimated: configural, metric, and scalar (Byrne and Matsumoto, 2021). They are defined by the parameters that are constrained to be equal across both samples (Milfont and Fischer, 2010; Beaujean, 2014). In the baseline model (i.e., configural invariance), no equality constraints were made—this allowed us to determine if the factor structures were the same across both samples. Only when configural invariance was established, metric invariance was estimated—by constraining factor loadings to be equal across both groups. Similarly, only when metric invariance was established, scalar invariance was estimated—by constraining intercepts to be equal across both groups. Cut-off criteria as recommended by Chen (2007) was used to identify levels of measurement invariance: ΔCFI ≤ 0.01.

We next assessed the assumption of normality for all variables—RFP, AFP, individualism, collectivism, attitude toward caring for elderly parents. Values for skewness and kurtosis for all variables were within the acceptable standards for a normal distribution, that is, between −2 and +2 (George and Mallery, 2010). Univariate outliers were also identified for RFP (*N* = 10), AFP (*N* = 2), individualism (*N* = 6), collectivism (*N* = 1), and attitude toward caring for elderly parents (*N* = 6). To deal with these issues, we excluded cases bearing the univariate outliers; this left us with a sample of 252 participants. Subsequent analyses were conducted with and without these univariate outliers.

Further, as we were interested to examine difference in RFP and AFP across cultures, we planned to conduct an independent *t*-test to compare the scores of RFP and AFP across Asian Americans and Caucasian Americans. We also intended to conduct multiple regression analyses to test the effects of RFP and AFP on predicting attitude toward caring for elderly parents and filial behaviors in both Asian Americans and Caucasian Americans. Gender, age, occupational status (coded as 1 = working full time, 0 = not working full-time), primary caregiver status (coded as 0 = no, 1 = yes), and number of parents who are still alive were included in the regression model as control variables (Yeh et al., 2013). Prior to conducting the multiple regression analyses, statistical assumptions relevant to multiple regression analysis—that is, normality, linearity and homoscedasticity of residuals, and multicollinearity between predictors—were examined, and no assumptions violations were noted. Mahalanobis distance exceeded for the critical χ^2^ for df = 7 (at α = 0.001) of 24.32 for two cases in the data file for the dependent variable of filial attitude, and two cases for the dependent variable of filial behaviors, indicating the presence of multivariate outliers. Multiple regression analysis and parallel mediation analysis were conducted with and without the case bearing the multivariate outlier.

Finally, to examine if the cultural dimension of collectivism is responsible for driving RFP and AFP in predicting care attitude toward parents and filial behaviors, parallel mediation analyses using PROCESS version 3.1 (Hayes, 2018) were conducted. For mediation to be demonstrated, the bootstrap confidence interval of the indirect effect (path a^*^b) must not include zero (bootstrap samples = 5,000) (Hayes, 2018). To rule out alternative explanations, we also conducted further tests to investigate if individualism and ethnicity would predict RFP and AFP to consequently influence filial attitude and behaviors.

## Results

### Measurement Invariance of the Scales Used

First, a series of CFAs was conducted—testing the two-factor model of the DFPS, the one-factor model of the attitude toward caring for elderly parents, and the two-factor model of the Culture Orientation Scale. As seen in Table 1, the CFI, RMSEA, and SRMR values suggested a good fit.

**Table 1:** CFA fit statistics for structural models of scales used in study.

**Measure**	**χ^2^**	***df***	**CFI**	**RMSEA**	**SRMR**
Dual filial piety scale	262.977	97	0.949	0.079	0.077
Attitude toward caring for elderly parents	209.03	100	0.955	0.063	0.052
Culture orientation scale	157.084	85	0.949	0.055	0.063

*χ^2^, chi square; df, degrees of freedom; CFI, comparative fit index; RMSEA root mean square error of approximation; SRMR, standardized root mean square residual*.

Next, a three-level MI test was conducted for each scale. Table 2 shows the global fit coefficients for the three levels of MI (configural, metric, scalar) for each scale. As seen, the three measures have reached scalar invariance across both samples—indicating that samples from both cultures understood the meaning of the latent construct of filial piety, caring toward elderly parents, and individualism and collectivism—which would allow us to make cross-cultural comparisons.

**Table 2:** Global fit measures in measurement invariance tests for scales used in study.

**Measure**	**Level of invariance**	**χ^2^**	***df***	**CFI**	**ΔCFI**
Dual filial piety scale	Configural (equal form)	405.27	194	0.935	-
	Metric (equal factor loadings)	415.41	208	0.936	0.001
	Scalar (equal intercepts)	442.46	222	0.933	0.004
Attitude toward caring for elderly parents	Configural (equal form)	350.23	200	0.940	-
	Metric (equal factor loadings)	365.22	216	0.940	0.000
	Scalar (equal intercepts)	378.97	232	0.941	0.001
Culture orientation scale	Configural (equal form)	250.32	170	0.945	-
	Metric (equal factor loadings)	266.75	184	0.943	0.002
	Scalar (equal intercepts)	289.91	198	0.937	0.006

*χ^2^, chi square; df, degrees of freedom; CFI, comparative fit index; ΔCFI, change in CFI*.

### Hypothesis Testing

Table 3 displays the means, standard deviations, skewness, kurtosis, and intercorrelations of all the variables involved in this study. Correlation analysis indicated that collectivism was positively associated with both RFP (*r* = 0.49, *p* < 0.01) and AFP (*r* = 0.23, *p* < 0.01). Individualism was positively correlated only with RFP (*r* = 0.14, *p* = 0.03), but not AFP (*r* = 0.10, *p* = 0.10). Both RFP (*r* = 0.40, *p* < 0.01) and AFP (*r* = 0.33, *p* < 0.01) were positively associated with attitude toward caring for one's elderly parents. RFP was positively associated with filial behaviors (*r* = 0.21, *p* < 0.01), but not AFP (*r* = 0.08, *p* = 0.27). RFP and AFP are not significantly correlated to each other (*r* = 0.05, *p* = 0.42). These patterns of findings are reflected in both Asian Americans and Caucasian Americans samples (see Table 4).

**Table 3:** Descriptive statistics and intercorrelations of all variables (*N* = 252).

**Variables**	**1**.	**2**.	**3**.	**4**.	**5**.	**6**.
1. RFP	-					
2. AFP	0.05	-				
3. Individualism	0.14*	0.10	-			
4. Collectivism	0.49**	0.23**	0.19*	-		
5. Filial attitude	0.40**	0.33*	−0.02	0.36**	-	
6. Filial behavior	0.21**	0.08	−0.14	0.17*	0.18*	-
Mean	5.78	3.29	5.78	6.41	2.75	−0.14
SD	0.96	1.17	0.97	1.28	0.44	0.79
Skew	−0.80	0.28	0.01	−0.20	0.06	0.24
Kurtosis	−0.18	−0.66	−0.22	−0.44	021	−0.50

** *Correlation significant at p < 0.01*.

* *Correlation significant at p < 0.05*.

**Table 4:** Descriptive statistics and intercorrelations of all variables across Caucasian and Asian samples (*N* = 252).

**Variables**	**Caucasian American (*****N*** **= 141)**						**Asian American (*****N*** **= 111)**					
	**1**.	**2**.	**3**.	**4**.	**5**.	**6**.	**1**.	**2**.	**3**.	**4**.	**5**.	**6**.
1. RFP	-						-					
2. AFP	−0.04	-					0.16	-				
3. Individualism	0.10	0.07	-				0.18	0.13	-			
4. Collectivism	0.46**	0.17*	0.16	-			0.53**	0.30**	0.23*	-		
5. Filial attitude	0.35**	0.34**	0.06	0.34**	-		0.46**	0.32**	−0.12	0.38**	-	
6. Filial behavior	0.20*	0.09	−0.21*	0.16	0.17	-	0.27**	0.15	0.08	0.22	0.24	-
Mean	5.81	3.10	5.73	6.38	2.75	−0.10	5.76	3.54	5.83	6.44	2.75	−0.21
SD	0.90	1.13	0.96	1.32	0.45	0.88	1.04	1.18	0.98	1.22	0.43	0.60
Skew	−0.77	0.39	0.20	−0.24	−0.08	0.10	−0.80	−0.08	−0.21	−0.14	0.26	0.54
Kurtosis	0.17	−0.37	−0.15	−0.38	0.14	−0.82	−0.28	−0.70	−0.18	−0.55	0.36	0.12

** *Correlation significant at p < 0.01*.

* *Correlation significant at p < 0.05*.

An independent *t*-test was conducted to examine levels of RFP and AFP between Asian Americans and Caucasian Americans. Results revealed that there was no significant difference in RFP between both groups, *t*_(250)_ = 0.41, *p* = 0.68*, d* = 0.05. There was, however, a significant difference in AFP between Asian Americans and Caucasian Americans, *t*_(250)_ = −2.96, *p* < 0.01, *d* = 0.38, where AFP was higher for Asian Americans (*M* = 3.54, *SD* = 1.18) than Caucasian Americans (*M* = 3.10, *SD* = 1.13). A sensitivity analysis conducted using G-Power indicated that given sample size of group 1 (Asian) is 115 and the sample size of group 2 (Caucasians) is 138, the minimum effect size to detect a power of 0.80 at α = 0.05 (two-tailed) is *d* = 0.36 for this study.

A multiple regression analysis was conducted to examine the effect of RFP and AFP on attitude toward caring for one's elderly parents. On step 1 of the hierarchical multiple regression analysis, age, gender, occupation/job status, primary caregiver status, and number of parents who are alive accounted for a significant 6.6% of the variance in attitude toward caring for elderly parents, *R*^2^ = 0.07, *F*_(5,244)_ = 3.45, *p* < 0.01. On step 2, RFP and AFP were added to the regression equation and accounted for an additional 22.2% of the variance in the attitude toward caring for one's elderly parents, Δ*R*^2^ = 0.22, Δ*F*_(2,242)_ = 37.71, *p* < 0.01. In combination, the six predictor variables explained 28.8% of the variance in attitude toward caring for elderly parents, *R*^2^ = 0.29, adjusted *R*^2^ = 0.27, *F*_(7,242)_ = 13.98, *p* < 0.01, *f*^2^ = 0.40. The analysis revealed that both RFP, *B* = 0.16, *t*_(244)_ = 6.21, *p* < 0.01, 95% CI (0.11, 0.21), and AFP, *B* = 0.12, *t*_(244)_ = 5.44, *p* < 0.01, 95% CI (0.07, 0.16), predicted attitude toward caring for elderly parents^4^. This regression model was also examined using Asian Americans and Caucasian Americans samples separately. The results showed that the model explained 36.0% of the variance in attitude toward caring for elderly parents in the Asian Americans sample (*R*^2^ = 0.36, adjusted *R*^2^ = 0.32, *F*_(7,102)_ = 8.21, *p* < 0.01, *f*^2^ = 0.56) and 29.6% of the variance in the Caucasian Americans sample [*R*^2^ = 0.30, adjusted *R*^2^ = 0.26, *F*_(7,132)_ = 7.92, *p* < 0.01, *f*^2^ = 0.42]. RFP and AFP predicted attitudes toward caring for elderly parents in both Asian Americans and Caucasian Americans samples. Unstandardized (*B*) regression coefficients for each predictor are reported in Table 5.

**Table 5:** Unstandardized (B) regression coefficients for multiple regression model predicting filial attitude.

**Variables**	**Overall (*****N*** **= 250)**				**Caucasian Americans (*****N*** **= 140)**				**Asian Americans (*****N*** **= 110)**			
	***B***	**LLCI**	**ULCI**	**se**	***B***	**LLCI**	**ULCI**	**se**	***B***	**LLCI**	**ULCI**	**se**
**Step 1**												
Gender	0.02	−0.09	0.13	0.06	0.07	−0.10	0.23	0.08	−0.04	−0.19	0.11	0.08
Age	0.01	>0.001	0.01	0.002	0.01	−0.001	0.01	0.003	0.01*	0.001	0.01	0.003
Occupation status	−0.03	−0.14	0.08	0.06	0.11	−0.05	0.26	0.08	−0.21*	−0.38	−0.05	0.08
Primary caregiver	0.19**	0.06	0.32	0.06	0.09	−0.09	0.27	0.09	0.25**	0.07	0.43	0.09
Parents	−0.02	−0.12	0.08	0.05	−0.002	−0.13	0.12	0.06	−0.03	−0.19	0.13	0.08
**Step 2**												
Gender	0.04	−0.06	0.14	0.05	0.10	−0.06	0.25	0.08	−0.02	−0.15	0.12	0.07
Age	0.004	−0.001	0.01	0.002	0.01	−0.001	0.01	0.003	0.004	−0.002	0.01	0.003
Occupation status	0.02	−0.08	0.12	0.05	0.13	−0.01	0.26	0.07	−0.13	−0.28	0.02	0.08
Primary caregiver	0.12*	0.004	0.23	0.06	0.01	−0.15	0.16	0.08	0.22**	0.06	0.38	0.08
Parents	−0.01	−0.09	0.08	0.04	0.02	−0.10	0.13	0.06	0.01	−0.14	0.15	0.07
RFP	0.16**	0.11	0.21	0.03	0.16**	0.09	0.24	0.04	0.16**	0.09	0.23	0.04
AFP	0.12**	0.07	0.16	0.02	0.15**	0.09	0.21	0.03	0.07*	0.01	0.13	0.03

* *p < 0.05*.

** *p < 0.01*.

A similar multiple regression analysis was conducted to examine the effect of RFP and AFP on filial behaviors. Results revealed that the seven predictor variables explained 43.8% of the variance in filial behaviors, *R*^2^ = 0.44, adjusted *R*^2^ = 0.42, *F*_(7,171)_ = 19.04, *p* < 0.01, *f*^2^ = 0.78. The results also revealed that RFP, *B* = 0.18, *t*_(171)_ = 3.60, *p* < 0.01, 95% CI (0.08, 0.28), predicted filial behaviors, but not AFP, *B* = −0.02, *t*_(171)_ = −0.43, *p* = 0.67, 95% CI (−0.10, 0.06). This regression model was also examined using Asian Americans and Caucasian Americans samples separately. The results showed that the model explained 38.0%% of the variance in filial behaviors in the Asian Americans sample [*R*^2^ = 0.38, adjusted *R*^2^ = 0.31, *F*_(7,59)_ = 2.92, *p* < 0.01, *f*^2^ = 0.61] and 52.4% of the variance in the Caucasian Americans sample [*R*^2^ = 0.54, adjusted *R*^2^ = 0.49, *F*_(7,104)_ = 16.35, *p* < 0.01, *f*^2^ = 1.17]. RFP, but not AFP, predicted filial behaviors in both samples. Unstandardized (*B*) regression coefficients for each predictor are reported in Table 6. A sensitivity analysis conducted using G-Power indicated that given a total sample size of 250, the minimum effect size to detect a power of 0.80 at α = 0.05 is *f*^2^ = 0.06 for this study.

**Table 6:** Unstandardized (B) regression coefficients for multiple regression model predicting filial behaviors.

**Variables**	**Overall (***N =*** 179)**				**Caucasian Americans (***N =*** 112)**				**Asian Americans (***N*** = 67)**			
	***B***	**LLCI**	**ULCI**	**se**	***B***	**LLCI**	**ULCI**	**se**	***B***	**LLCI**	**ULCI**	**se**
**Step 1**												
Gender	0.04	−0.16	0.24	0.10	0.04	−0.23	0.31	0.14	0.02	−0.26	0.30	0.14
Age	0.003	−0.01	0.01	0.004	0.002	−0.01	0.01	0.01	0.002	−0.01	0.01	0.01
Occupation status	−0.03	−0.22	0.16	0.10	−0.02	−0.27	0.23	0.13	−0.12	−0.40	0.16	0.14
Primary caregiver	1.01**	0.75	1.27	0.13	1.23**	0.86	1.61	0.19	0.79**	0.45	1.12	0.17
Parents	0.46**	0.30	0.61	0.08	0.53**	0.34	0.73	0.10	0.27*	0.004	0.53	0.13
**Step 2**												
Gender	−0.003	−0.20	0.19	0.10	−0.002	−0.28	0.28	0.14	0.06	−0.21	0.34	0.14
Age	0.001	−0.01	0.01	0.004	0.001	−0.01	0.01	0.01	−0.001	−0.01	0.01	0.01
Occupation status	0.01	−0.18	0.20	0.09	−0.03	−0.27	0.22	0.12	0.03	−0.27	0.32	0.15
Primary caregiver	0.96**	0.71	1.22	0.13	1.17**	0.80	1.55	0.19	0.80**	0.47	1.13	0.17
Parents	0.47**	0.32	0.62	0.08	0.54**	0.35	0.73	0.10	0.29*	0.03	0.54	0.13
RFP	0.18**	0.08	0.28	0.05	0.18*	0.03	0.33	0.07	0.16*	0.03	0.29	0.07
AFP	−0.02	−0.10	0.06	0.04	0.05	−0.07	0.17	0.06	−0.003	−0.12	0.11	0.06

* *p < 0.05*.

** *p < 0.01*.

**Figure 1:**
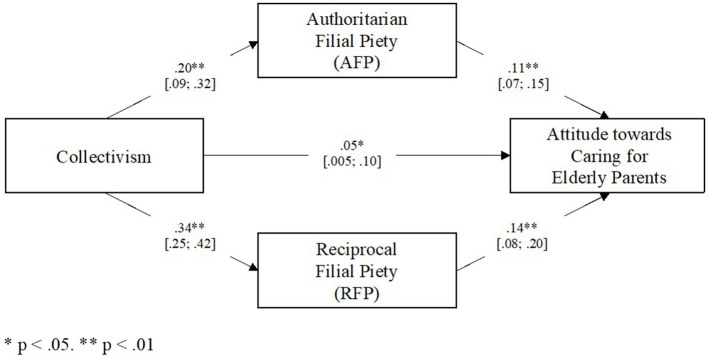
Parallel Mediation Model with path estimates and 95% Confidence Interval. The following variables were included as covariates: individualism, ethnicity, gender, age, occupational status, primary caregiver status, number of parents alive. *p < 0.05; **p < 0.01.

A parallel mediation analysis using PROCESS (Hayes, 2018) was conducted to examine if collectivism predict RFP and AFP, which consequently predict attitude toward caring for elderly parents. Figure 1 displays the mediation model. Both RFP and AFP were included as mediators in the analysis. Results revealed that there was a direct effect of collectivism on filial attitudes, *B* = 0.05, *p* = 0.03, 95% CI (0.005, 0.10). Results also showed that collectivism predicted RFP, *B* = 0.34, *p* < 0.01, 95% CI (0.25, 0.42), and AFP, *B* = 0.20, *p* < 0.01, 95% CI (0.09, 0.32). Next, results also revealed that both RFP, *B* = 0.14, *p* < 0.01, 95% CI (0.08, 0.20), and AFP, *B* = 0.11, *p* < 0.01, 95% CI (0.07, 0.15), predicted attitude toward caring for elderly parents. Finally, results indicated that collectivism predicted positive attitude toward caring for elderly parents *via* both RFP, *B* = 0.05, 95% CI = (0.03, 0.07), and AFP, *B* = 0.02, 95% CI = (0.01, 0.04)^5^. Unstandardized (*B*) regression coefficients, 95% confidence intervals, and *R*^2^-values for the parallel mediation model are presented in Table 7.

**Table 7:** Parallel mediation model coefficients for predicting filial attitude.

**Variables**	**Overall (*****N*** **= 250)**				**Caucasians (*****N*** **= 140)**				**Asians (*****N*** **= 110)**			
**Outcome Variable = RFP**	***B***	**LLCI**	**ULCI**	**se**	***B***	**LLCI**	**ULCI**	**se**	***B***	**LLCI**	**ULCI**	**se**
	**(***R***^2^ = 0.30, ***p*** < 0.01)**				**(***R***^2^ = 0.28, ***p*** < 0.01)**				**(***R***^2^ = 0.34, ***p*** < 0.01)**			
Constant	2.80	1.83	3.78	0.50	2.90	1.65	4.15	0.63	2.71	1.09	4.34	0.82
Collectivism	0.34**	0.25	0.42	0.04	0.28**	0.18	0.39	0.05	0.40**	0.25	0.54	0.07
Individualism	0.07	−0.04	0.18	0.06	0.05	−0.09	0.19	0.07	0.09	−0.08	0.27	0.09
Ethnicity	−0.02	−0.24	0.20	0.11	-	-	-	-	-	-	-	-
Gender	0.18	−0.03	0.40	0.11	0.28	−0.01	0.57	0.15	0.11	−0.23	0.45	0.11
Age	0.01	−0.001	0.02	0.005	0.01	−0.002	0.02	0.006	0.01	−0.01	0.02	0.01
Occupational status	−0.28*	−0.50	−0.07	0.11	−0.22	−0.50	0.06	0.14	−0.34	−0.71	0.03	−0.34
Primary caregiver	0.04	−0.20	0.28	0.12	0.05	−0.27	0.37	0.16	−0.03	−0.42	0.36	−0.03
No of parents alive	−0.05	−0.24	0.13	0.09	0.01	−0.21	0.23	0.11	−0.19	−0.55	0.16	−0.19
**Outcome variable = AFP**	**(***R***^2^ = 0.16, ***p*** < 0.01)**				**(***R***^2^ = 0.14, ***p*** < 0.01)**				**(***R***^2^ = 0.15, ***p*** < 0.01)**			
Constant	2.40	1.09	3.71	0.67	3.46	1.73	5.19	0.87	1.37	−0.73	3.47	1.06
Collectivism	0.20**	0.09	0.31	0.06	0.17*	0.03	0.32	0.07	0.27**	0.08	0.45	0.10
Individualism	0.07	−0.07	0.22	0.07	0.05	−0.14	0.25	0.10	0.09	−0.14	0.31	0.11
Ethnicity	0.31*	0.02	0.60	0.15	-	-	-	-	-	-	-	-
Gender	−0.38**	−0.67	−0.10	−0.38	−0.61**	−1.02	−0.21	0.20	−0.13	−0.57	0.30	0.22
Age	−0.01*	−0.02	−0.0003	−0.01	−0.01	−0.03	0.002	0.01	−0.003	−0.02	0.02	0.01
Occupational status	−0.06	−0.35	0.23	−0.06	−0.04	−0.42	0.34	0.19	−0.07	−0.55	0.41	0.24
Primary caregiver	0.44**	0.12	0.77	0.16	0.41	−0.03	0.85	0.22	0.57	0.06	1.07	0.25
No of parents alive	−0.003	−0.25	0.24	0.13	−0.09	−0.40	0.22	0.16	0.10	−0.36	0.56	0.23
**Outcome variable = filial attitudes**	**(***R***^2^ **= 0.31**, ***p*** < 0.01)**				**(***R***^2^ **= 0.31**, ***p*** < 0.01)**				**(***R***^2^ **= 0.43**, ***p*** < 0.01)**			
Constant	1.38	0.89	1.86	0.25	0.89	0.18	1.60	0.36	1.79	1.13	2.46	0.34
Collectivism	0.05*	0.005	0.09	0.02	0.04	−0.02	0.10	0.03	0.06	−0.001	0.12	0.03
RFP	0.14**	0.08	0.20	0.03	0.14**	0.06	0.23	0.04	0.14**	0.07	0.22	0.04
AFP	0.11**	0.07	0.15	0.02	0.15**	0.08	0.21	0.03	0.07*	0.01	0.13	0.03
Individualism	−0.06*	−0.11	−0.01	0.03	−0.02	−0.09	0.05	0.04	−0.11	−0.17	−0.04	0.03
Ethnicity	0.003	−0.10	0.10	0.05	-	-	-	-	-	-	-	-
Gender	0.04	−0.06	0.13	0.04	0.09	−0.06	0.24	0.08	−0.005	−0.14	0.13	0.07
Age	0.003	−0.001	0.01	0.003	0.01	−0.001	0.01	0.003	0.004	−0.002	0.01	0.003
Occupational status	0.03	−0.07	0.13	0.03	0.12	−0.01	0.26	0.07	−0.09	−0.24	0.06	0.07
Primary caregiver	0.10*	−0.01	0.22	0.06	−0.003	−0.16	0.16	0.08	0.21**	0.06	0.37	0.08
No of parents alive	−0.01	−0.09	0.07	0.04	0.02	−0.09	0.13	0.06	0.001	−0.14	0.14	0.07

* *p < 0.05*.

** *p < 0.01*.

A similar parallel mediation analysis was conducted with individualism as the independent variable. Individualism had a direct effect on attitude toward caring for elderly parents, *B* = −0.06, *p* = 0.02, 95% CI (−0.11, 0.01). Individualism did not predict RFP, *B* = 0.07, *p* = 0.18, 95% CI (−0.04, 0.18), and AFP, *B* = 0.07, *p* = 0.33, 95% CI (−0.07, 0.22). Individualism did not predict attitude toward caring for elderly parents *via* both RFP, *B* = 0.01, 95% *CI* = (−0.01, 0.03), and AFP, *B* = 0.01, 95% *CI* = (−0.01, 0.03).

When ethnicity (i.e., Asian Americans and Caucasian Americans) was included as the independent variable, the results showed that only AFP mediated the relationship between ethnicity and attitude toward caring for elderly parents. Specifically, ethnicity predicted AFP, *B* = 0.31, *p* = 0.04, 95% CI (0.02, 0.60), but not RFP, *B* = −0.02, *p* = 0.88, 95% CI (−0.24, 0.20). Identifying with an Asian identity predicted attitude toward caring for elderly parents *via* AFP, *B* = 0.03, 95% *CI* = (0.001, 0.07), and not RFP, *B* = −0.003, 95% *CI* = (−0.03, 0.03). There was also no direct effect of ethnic group on attitude toward caring for elderly parents, *B* = −0.003, *p* = 0.96, 95% CI (−0.10, 0.10).

Similar parallel mediation analysis was conducted to examine if collectivism predict RFP and AFP, which consequently predict filial behaviors. Results revealed that RFP predicted filial behaviors, *B* = 0.17, *p* < 0.01, 95% CI (0.06, 0.28), but not AFP, *B* = 0.02, *p* = 0.66, 95% CI (−0.06, 0.10). Furthermore, collectivism predicted filial behaviors *via* RFP, *B* = 0.06, 95% CI (0.02, 0.10), but not AFP, *B* = 0.002, 95% CI (−0.01, 0.02)^6^. Unstandardized (*B*) regression coefficients, 95% confidence intervals, and *R*^2^-values for the parallel mediation model are presented in Table 8. Further parallel mediation analysis also showed that individualism had a direct effect on filial behaviors, *B* = −0.10, *p* = 0.03, 95% CI (−0.19, −0.01), but this effect was not mediated by RFP [*B* = 0.02, 95% CI (−0.01, 0.05)] nor AFP [*B* = 0.001, 95% CI (−0.01, 0.01)]. Finally, the results of a similar parallel mediation analysis revealed that ethnicity had a significant effect on filial behaviors directly [*B* = −0.31, *p* < 0.01, 95% CI (−0.51, 0.12)], but this was not mediated through RFP [*B* = −0.02, 95% CI (−0.07, 0.03)] and AFP [*B* = 0.01, 95% CI (−0.03, 0.07)].

**Table 8:** Parallel mediation model coefficients for predicting filial behaviors.

**Variables**	**Overall (***N*** = 179)**				**Caucasians (***N*** = = 112)**				**Asian (***N*** = = 67)**			
**Outcome variable = RFP**	***B***	**LLCI**	**ULCI**	**se**	***B***	**LLCI**	**ULCI**	**se**	***B***	**LLCI**	**ULCI**	**se**
	**(***R***^2^ **= 0.32**, ***p*** < 0.01)**				**(***R***^2^ **= 0.31**, ***p*** < 0.01)**				**(***R***^2^ **= 0.39**, ***p*** < 0.01)**			
Constant	2.46	1.22	3.69	0.63	2.78	1.38	4.17	0.71	2.20	−0.38	4.77	1.29
Collectivism	0.35**	0.25	0.44	0.05	0.31**	0.20	0.42	0.06	0.34**	−0.14	0.54	0.10
Individualism	0.10	−0.02	0.23	0.10	0.05	−0.09	0.20	0.07	0.19	−0.06	0.44	0.19
Ethnicity	−0.09	−0.35	0.17	−0.09	-	-	-	-	-	-	-	-
Gender	0.23	−0.02	0.49	0.13	0.32*	0.01	0.63	−0.16	0.07	−0.43	0.57	0.25
Age	0.01*	−0.004	0.02	0.01	0.01	−0.01	−0.02	0.01	0.02	−0.003	0.04	0.01
Occupational status	−0.26	−0.51	−0.02	0.17	−0.11	−0.41	0.18	0.15	−0.55*	−1.07	−0.03	0.26
Primary caregiver	0.13	−0.21	0.47	0.10	0.20	−0.25	0.64	0.22	−0.10	−0.67	0.47	0.29
No. of parents alive	−0.03	−0.23	0.17	0.06	−0.004	−0.23	0.22	0.12	−0.18	−0.63	0.27	0.22
**Outcome variable = AFP**	**(***R***^2^ **= 0.15**, ***p*** < 0.01)**				**(***R***^2^ **= 0.08**, ***p*** < 0.01)**				**(***R***^2^ **= 0.15**, ***p*** < 0.01)**			
Constant	2.75	1.09	4.42	0.84	3.87	1.89	5.85	1.00	0.56	−2.68	3.79	1.62
Collectivism	0.15*	0.02	0.27	0.06	0.11	−0.05	0.27	0.08	0.24	−0.01	0.50	0.13
Individualism	0.03	−0.14	0.21	0.09	−0.06	−0.25	0.15	0.11	0.16	−0.15	0.48	0.16
Ethnicity	0.56**	0.21	0.90	0.18	-	-	-	-	-	-	-	-
Gender	−0.31	−0.66	0.04	0.18	−0.52*	−0.96	−0.08	0.22	0.18	−0.45	0.81	0.32
Age	−0.01	−0.02	0.01	0.01	−0.01	−0.03	0.01	0.01	−0.01	−0.03	0.02	0.01
Occupational status	−0.17	−0.50	0.16	0.17	−0.05	−0.47	0.36	0.21	−0.05	−0.71	0.60	0.33
Primary caregiver	0.13	−0.32	0.59	0.23	0.13	−0.75	0.50	0.32	0.50	−0.22	1.22	0.36
No. of parents alive	0.07	−0.20	0.34	0.14	0.03	−0.30	0.35	0.16	0.23	−0.34	0.79	0.28
**Outcome variable = filial behaviors**	**(***R***^2^ **= 0.49**, ***p*** < 0.01)**				**(***R***^2^ **= 0.55**, ***p*** < 0.01)**				**(***R***^2^ **= 0.38**, ***p*** < 0.01)**			
Constant	−1.57	−2.53	−0.62	0.48	−1.59	−2.90	−0.28	0.66	−1.94	−3.46	−0.41	0.76
Collectivism	0.03	−0.05	0.11	0.04	0.03	−0.07	0.14	0.05	0.03	−0.10	0.16	0.06
RFP	0.17**	0.06	0.28	0.06	0.17*	0.004	0.33	0.08	0.15	−0.01	0.30	0.07
AFP	0.02	−0.06	0.10	0.04	0.04	−0.08	0.16	0.06	−0.01	−0.13	0.11	0.06
Individualism	−0.10*	−0.19	−0.01	0.05	−0.14*	−0.26	−0.01	0.06	−0.01	−0.16	0.14	0.07
Ethnicity	−0.31**	−0.51	−0.12	0.10	-	-	-	-	-	-	-	-
Gender	0.02	−0.17	0.21	0.10	−0.01	−0.28	0.27	0.14	0.09	−0.20	0.38	0.14
Age	−0.001	−0.01	0.01	0.004	0.002	−0.01	0.01	0.01	−0.002	−0.01	0.01	0.01
Occupational status	0.03	−0.16	0.21	0.09	0.03	−0.22	0.27	0.12	0.05	−0.27	0.36	0.16
Primary caregiver	0.98**	0.74	1.23	0.13	1.10**	0.73	1.48	0.19	0.80**	−0.47	1.14	0.17
No. of parents alive	0.48**	0.34	0.63	0.07	0.55**	0.36	0.74	0.10	0.28	0.02	0.54	0.13

* *p < 0.05*.

** *p < 0.01*.

## Discussion

We began our research with the aim of understanding the relationship between filial piety and attitude toward caring for elderly parents across cultures. Using the DFPM framework, we hypothesized that RFP, which develops from positive interactions between parent and child, is likely to be universal across cultures while AFP, which is guided by traditional Confucius notions of filial piety, is likely to be different across Asian and Western cultures. Consistent with what we have predicted, we found that AFP was higher in Asian Americans than Caucasian Americans in the United States, suggesting a cultural difference in AFP. This finding lent support for AFP as an indigenous Asian conceptualization of filial piety based on Confucius teachings. In contrast, there was no difference in RFP between both Asian Americans and Caucasian Americans, which suggests cultural universality. This finding provided empirical evidence for the notion of DFPM as a theoretical model that captures filial piety in global context (Bedford and Yeh, 2019). Additionally, our findings demonstrated that both RFP and AFP predicted filial attitudes whereas only RFP predicted filial piety behaviors. Finally, we ascertained that collectivism, rather than one's ethnicity, is the underlying dimension behind RFP and AFP, which consequently predicted filial attitudes.

The findings of our paper provide empirical support for cultural universality and difference between Asian Americans' and Caucasian Americans' filial piety in the United States. While filial piety is considered a salient Asian virtue and is predominantly observed in Asian societies, our findings demonstrated that caring for one's elderly parents is also practiced in Western societies, which consistent with the universal affect one would have toward their parents. In support for Bedford and Yeh (2019) proposal, the over-emphasis on AFP aspects of filial piety—that largely relies on Asian conceptualizations of filial piety—has limited the understanding of filial piety in cross-cultural context. Different from AFP, which is chiefly based on a hierarchical relationship between family roles, RFP is grounded on an equal and reciprocating relationship between parents and children, and as such, RFP can afford the study of filial piety in a globalized context (Bedford and Yeh, 2019). Our findings provide initial empirical evidence for the validation of the two-factor DFPM as a framework to study filial piety in cross-cultural context and to tease apart cultural similarities and differences in filial piety constructs, attitudes, and behaviors. Future research can apply DFPM to various cultural context to test the boundary of RPF and APF in different cultural societies.

Furthermore, our work provides support for DFPM in predicting filial attitudes and behaviors. RFP and AFP were both associated with more positive attitude toward providing care for elderly parents, which were consistent with the notion that both RFP and AFP lead to positive filial outcomes (Bedford and Yeh, 2019). Similarly, our study revealed that RFP had a stronger influence than AFP in predicting filial attitudes and behaviors, which is consistent with existing findings and supports the idea that RFP has broader and greater effects on various support and care behaviors (Yeh, 2009; Yeh et al., 2013). Additionally, we also found that RFP predicted filial behaviors, but not AFP. This is consistent with Yeh (2009) findings where AFP was correlated with emotional support of parents weakly.

Beyond supporting DFPM in the prediction of filial attitudes and behaviors, our work extends empirical work by demonstrating that the DFPM can be applied to non-Asian populations to study filial attitudes and behaviors. Our findings showed that RFP and AFP predicted adult children's attitude toward caring for their elderly parents, and RFP predicted filial behaviors, in both Asian Americans and Caucasian Americans samples, supporting the notion that filial piety is not unique to Asian cultures. Given different cultural practices in caregiving of elderly parents, it is intuitive to expect that RFP and AFP impact filial attitudes and behaviors differently across cultures. For instance, providing financial support is characteristic among the Chinese due to teachings such as “raising children for support in the old age (養兒防老),” which is incongruent with the ideology and norms in Western societies. Existing studies have also shown that elderly parents in Korea continue to receive financial support from their adult children as they age; in contrast, parents in Europe (e.g., Italy) were found to be financially independent (Deindl and Brandt, 2011; Floridi, 2019). As such, while we expected AFP to influence filial attitudes in Asians and not in Caucasians, our findings paint a different picture, in that AFP impacted filial attitudes in both Asian Americans and Caucasian Americans. Even though filial piety is not emphasized in Western societies, some evidence shows that individuals from more individualistic cultural backgrounds, such as Caucasian Americans, feel obligated to fulfill familial duties, including providing assistance to their family members (Freeberg and Stein, 1996). This suggests that AFP's influence on filial attitudes among Caucasians may be more pronounced than what has been assumed in existing literature. However, much contextual variation has been noted in Americans when it comes to obligatory feelings to elderly parents, such as the available care policies provided by the state they reside in and if care and support is dire (Cooney and Dykstra, 2011). Thus, future research is required to further understand the role of AFP in a Western cultural context.

Most importantly, rather than ethnic group difference, the findings showed that the endorsement of collectivism was associated with different levels of RFP and AFP, which in turn influenced filial piety attitudes and behaviors. Recent research has revealed a positive relationship between collectivism and willingness of taking care of elderly parents by “keeping them with us at home” (Talhelm, 2019). Going beyond the current findings, our research unveiled the differing impact of collectivism on RFP and AFP, as well as their downstream effect on filial piety attitudes and behaviors. Our method of untangling cultural influence corresponds to Masumoto's (1999) proposal that more important than observed cultural or national differences, researchers shall strive to pin down the underlying cultural dimensions such as values, norms, mindsets, and self-concepts that can explain the observable cultural and national difference. Future research can further investigate related psychological constructs of collectivism such as vertical and horizontal collectivism (Singelis et al., 1995) to explore potential impact of different types of collectivism on RFP and AFP as well as filial piety outcomes in different cultural context.

In sum, our study extends current literature by demonstrating the application of the DFPM framework to a non-Asian sample, and Asians living within a non-Asian context, which is distinct from existing studies that has applied the DFPM to across different Asian societies (Yeh et al., 2013). Such application, combined with the measurement of collectivism and individualism, further extends prior work by demonstrating that collectivistic values, rather than a person's ethnic group, underlies filial piety—both RFP and AFP aspects. Moreover, our results showed that being an Asian does not predict RFP, but being an Asian predicts AFP, which provides further support that AFP reflects the traditional indigenous Asian conceptualizations of filial piety.

### Limitations and Future Directions

The work we have presented here is far from conclusive and poses questions for future research. Firstly, in our work, we found that RFP was not correlated to AFP, which is inconsistent with existing findings that found a positive correlation between the two aspects of filial piety (Yeh et al., 2013). This is likely due to the sample we have employed in our study—that is, predominantly Caucasians—while previous research had been conducted using Asian samples. As such, this finding may imply that unlike Asians, where both aspects of filial piety are present, Caucasians, due to the absence of Confucian teaching in their culture, do not necessarily develop the AFP aspect of filial piety. Further, given that within the DFPM framework, there are four possible modes of personal interaction with parents (Yeh and Bedford, 2004)—that is the balanced mode (high RFP and high AFP), the reciprocal mode (high RFP and low AFP), the authoritarian mode (low RFP and high AFP), and the non-filial mode (low RFP and low AFP)—this lack of AFP development among Caucasians would have significant implications for the development of the different modes across cultures. Hence, further research is required to understand the development of AFP in non-Asian cultures to test the applicability of these four modes across cultures.

Secondly, one major limitation of this study is the sample we have employed in this study. In this study's sample, Asian Americans reported higher levels of individualism than Caucasian Americans, which contradicts the representations of Eastern (i.e., highly collectivistic) and Western (i.e., highly individualistic) cultures. Participants in this sample resided in the United States, which meant that for individuals who identified with being Asian, they were likely to either be first generation immigrants or second-generation Asian Americans. Studies have shown that first generation immigrants are likely to identify more with their home culture, and place greater emphasis of values originating from their home culture, more than their children (second generation) (e.g., Kunst and Sam, 2014; Stichnoth and Yeter, 2016). As such, the higher levels of individualism reported by Asians in this study could be an artifact of Asians in this study placing more emphasis of values from the host country (i.e., the United States). However, the data collected in this study limits us in ascertaining this as we did not measure the extent to which they acculturated to the host culture. In a similar vein, our findings also revealed that Asian participants reported lower frequencies of filial behaviors compared to the Caucasian participants. This could be confounded with the ease and convenience of engaging in filial behaviors. For Asian Americans participants, it is likely that their parents do not reside in the same country as them. Coupled with the fact that the data collection for this study was collected in the midst of the pandemic where travel across international borders is restricted, performing filial duties would be a challenge. As such, it is important to note that the findings presented in this study is preliminary and should be interpreted with caution. Additionally, we have only compared the differences within United states, including more points of comparisons (e.g., Caucasian Americans, Asian Americans, and Asians) would add further support to the results of this study. Future studies should include such changes to derive at more accurate conclusions about cross cultural differences in filial piety.

Lastly, as a single self-report questionnaire was used, common method variance (CMV) may be a concern. Future studies could avoid any potential CMV by using other sources of information for some of the key measures, including the perceived (vs. felt) level of filial piety of the adult children by their parents.

## Conclusion

This study aimed to examine the relationship between filial piety and attitude toward caring for elderly parents across cultures. Using the DFPM framework, we found that RFP does not differ across cultures while AFP does. We also found that collectivism, rather than ethnicity, predicted RFP and AFP, which consequently predicted positive attitude toward caring for elderly parents. Our work demonstrated the cross-cultural applicability of the DFPM model and have highlighted the universal and culture-specific aspects of filial piety.

## Data Availability Statement

The raw data supporting the conclusions of this article will be made available by the authors, without undue reservation.

## Ethics Statement

The studies involving human participants were reviewed and approved by Singapore Management University IRB. The patients/participants provided their written informed consent to participate in this study.

## Author Contributions

AL conceptualized the research idea, collected and analyzed the data, and drafted the manuscript. CL conducted initial data analyses and contributed to the literature review. C-YC reviewed the manuscript and provided important feedback for the final draft. All authors have read, edited, approved the final manuscript, and agree to be accountable for the content of this article.

## Conflict of Interest

The authors declare that the research was conducted in the absence of any commercial or financial relationships that could be construed as a potential conflict of interest.

## Publisher's Note

All claims expressed in this article are solely those of the authors and do not necessarily represent those of their affiliated organizations, or those of the publisher, the editors and the reviewers. Any product that may be evaluated in this article, or claim that may be made by its manufacturer, is not guaranteed or endorsed by the publisher.

## Footnotes

^1^Screening questions were included to ensure that participants identified with “Asian” or “Caucasian” as their ethnicity. We also included attentional checks to ensure the quality of data gathered from participants.

^2^Asian participants included individuals from Korea, China, Philippines, Vietnam, Japan, and India.

^3^Following Murray et al. (2005) recommendation to diminish the effects of common method bias, we have counterbalanced the order of the questions relating to different scales and constructs. Similarly, different scale endpoints and formats were also used to minimize method biases (Podsakoff et al., 2003).

^4^A similar regression analysis was conducted with the inclusion of univariate and multivariate outliers. The analysis including the outliers revealed similar findings. The model accounted for a significant 30.6% of the variability in filial attitude, *R*^2^ = 0.31, adjusted *R*^2^ = 0.29, *F*_(7,268)_ = 16.92, *p* < 0.01. The analysis revealed that RFP, *B* = 0.14, *t*_(268)_ = 6.41, *p* < 0.01, 95% CI (0.10, 0.18), and AFP, *B* = 0.11, *t*_(268)_ = 5.23, *p* < 0.01, 95% CI (0.07, 0.16), predicted attitude toward caring for elderly parents.

^5^A similar parallel mediation analysis was conducted without excluding univariate and multivariate outliers. Similar findings were found even with the inclusion of the outliers. Collectivism predicted attitude toward caring for elderly parents via RFP, *B* = 0.05, 95% *CI* = (0.02, 0.08), and AFP, *B* = 0.03, 95% *CI* = (0.01, 0.05).

^6^For filial behaviors, similar findings were found with the inclusion of the outliers. Collectivism predicted filial behaviors via RFP, *B* = 0.07, 95% *CI* = (0.03, 0.11), and not AFP, *B* = 0.01, 95% *CI* = (−0.01, 0.03).
